# Spontaneous pneumothorax: An unusual complication of pregnancy - A case report and review of literature

**DOI:** 10.4103/1817-1737.41915

**Published:** 2008

**Authors:** Rajiv Garg, Sanjay, Vinita Das, Kauser Usman, Sumit Rungta, R. Prasad

**Affiliations:** *Department of Pulmonary Medicine, King George's Medical University, Lucknow, India*; 1 *Department of Obstetrics and Gynecology, King George's Medical University, Lucknow, India*; 2 *Department of Medicine, King George's Medical University, Lucknow, India*

**Keywords:** Pneumothorax, pregnancy, spontaneous

## Abstract

Spontaneous pneumothorax complicating pregnancy is rare. Only 55 cases have been reported till now. We describe a case of a 30-year-old Indian woman with spontaneous pneumothorax during her 28^th^ week of pregnancy.

## Introduction

Dyspnea in a pregnant woman may arise as a result of underlying disease or the pregnancy itself. During pregnancy, pulmonary functional reserve, including functional residual capacity and total lung capacity, is decreased[1]; whereas oxygen consumption by the placenta, fetus, and maternal organs is increased.[1] In addition, physiological anemia of pregnancy and a relatively low partial pressure of oxygen in the umbilical vein of the fetus mean that any maternal hypoxic changes may not be tolerated.[2] Any impairment in ventilation during pregnancy may thus have serious consequences for both the mother and her fetus. Spontaneous pneumothorax complicating pregnancy is rare. We present a case of this rare entity during the third trimester that was treated conservatively.

## Case Report

A 30-year-old woman (gravida 3 para 2) at 28 weeks' gestation was admitted to the Emergency Department with complaint of chest pain (right side) followed by breathlessness. Breathlessness was sudden in onset and progressively worsened over 5 days. Chest pain was pleuritic in nature. She was also having history of exposure to household smoke. She was in mild respiratory distress, but her vital signs were stable while breathing room air. Her breath sounds were decreased with hyper resonance over her right chest.

**Figure 1:**
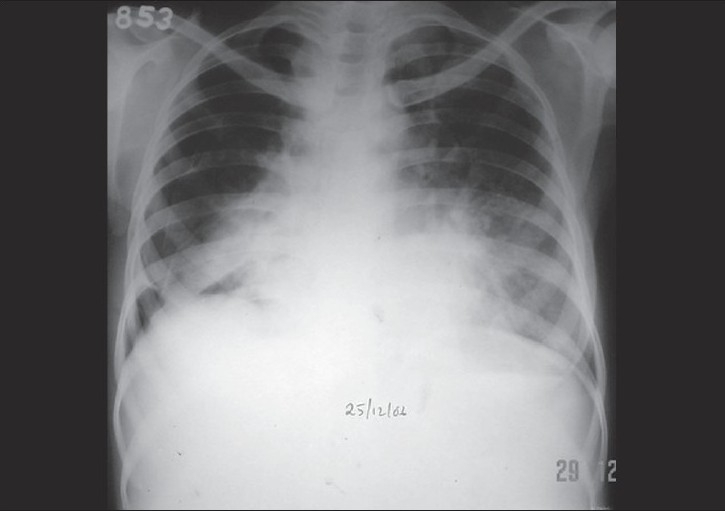
A chest radiograph showing right-sided pneumothorax

A chest radiograph with abdominal shield confirmed right-sided pneumothorax [Figure 1]. Results of other prenatal laboratory tests were normal. The patient was treated with oxygen and observed. After 10 days of conservative treatment, she recovered spontaneously. With supportive care, her condition improved and lung re-expansion was achieved without chest tube placement. A subsequent chest radiograph showed no evidence of residual pneumothorax, bullae, or any pulmonary pathology.

Ultrasound assessment revealed a singleton fetus in cephalic presentation, with fetal parameters corresponding to a gestational age of 28 weeks. The fetus was active and liquor was normal. She experienced no further chest pain, and the remainder of her pregnancy was uncomplicated. At 39 weeks' gestation, the patient underwent a spontaneous vaginal delivery of a viable female infant of weight 2.2 kg with good Apgar scores.

## Discussion

Primary spontaneous pneumothorax is defined as air in the pleural space, that is, between the lung and the chest wall in otherwise healthy people without any lung disease. Spontaneous pneumothorax in pregnancy is extremely rare, with only 55 cases reported till now.[1–8] Review of 56 cases (including one reviewed by the author) showed that the patients were young (average age, 26.4 years), which is similar to the age group (20-30 years) of nonpregnant female,[9] in whom pneumothorax commonly occurs. Risk factors most commonly associated in these patients were asthma, cocaine use, hyperemesis gravidarum, history of previous pneumothorax (44%), and underlying infection (30%); whereas pulmonary tuberculosis is the most common cause in nonpregnant females.[9] Pneumothorax occurred during the first or second trimester in 51% and during the perinatal period in 49% of patients. Initial treatment was observation in 29.6%, tube thoracostomy in 66.6%, and thoracotomy in 3.8% of patients. Of the total group of patients, 52% ultimately required thoracotomy for recurrence or persistent pneumothorax. The obstetric outcome was good, with 80.8% of patients having vaginal delivery, 17.3% having cesarean delivery, and one being fetal loss (1.9%). Typical pneumothorax symptoms such as chest pain and dyspnea are often attributed to paroxysmal tachycardia, neuralgia, or asthma exacerbation, thus contributing to underreporting of spontaneous pneumothorax.[10] Diagnosis of pneumothorax can be confirmed by chest radiograph, and it is safe to proceed with the standard chest radiography with abdominal shield without placing the fetus at substantial risk from ionizing radiation. Shielded computed tomography (CT) is also a useful imaging technique that can help in defining the underlying anatomic abnormality and in planning an operative approach when surgical treatment is indicated.[2]

Treatment of acute pneumothorax in pregnancy or labor is identical to that of non-obstetric patients. Admission and close observation of the patients was usually done with small pneumothorax (less than 20% of hemithorax).[11] Large pneumothorax (more than 20% of hemithorax) should be treated with tube thoracostomy. Recurrent, persistent, or bilateral pneumothorax necessitates thoracotomy or thoracoscopy. In order to avoid increased air leak secondary to valsalva maneuvers, delivery should be expedited and positive pressure anesthesia avoided.[2] Cesarean section is not absolutely indicated and should be performed for obstetric reason only.

Although surgery may be indicated for recurrent pneumothorax episodes, specific criteria for operative intervention are lacking. Thoracotomy or video assissted thoracoscopic surgery (VATS) have been increasingly successful in the management of recurrent pneumothorax, and no adverse outcome or mortality has been reported. Nevertheless, preventive measures should include smoking cessation and avoidance of rapid or drastic change in ambient pressure such as high altitudes, scuba diving, or flying in unpressurized aircraft.

Pneumothorax warrants consideration in any pregnant patient with acute chest pain, dyspnea, or history of prior pneumothorax and must be confirmed radiographically. Neither pneumothorax nor its treatment causes serious adverse effects on the course of pregnancy or delivery, but prompt recognition and treatment of pneumothorax is essential for preventing complications.
